# Metabolic and hormonal assessment of adolescent and young adult women with prior premature adrenarche

**DOI:** 10.6061/clinics/2019/e836

**Published:** 2019-06-11

**Authors:** Flávia Alves Ribeiro, Elisabete Aparecida Mantovani Rodrigues de Resende, Adriana Paula da Silva, Janaíne Machado Tomé, Heloísa Marcelina da Cunha Palhares, Maria de Fátima Borges

**Affiliations:** Divisao de Endocrinologia e Metabolismo, Universidade Federal do Triangulo Mineiro (UFTM), Uberaba, MG, BR

**Keywords:** Adrenarche, Polycystic Ovary Syndrome, Hirsutism, Glucose Metabolism Disorders, Dyslipidemias

## Abstract

**OBJECTIVE:**

Follow-up studies of girls with premature adrenarche have reported the development of polycystic ovary syndrome, insulin resistance, and dyslipidemia and a propensity to cardiovascular disease. The aim of this study was to analyze the presence of these conditions in patients previously treated at the Universidade Federal do Triângulo Mineiro.

**METHODS:**

A total of 130 medical records reported premature adrenarche. One hundred and twenty-two patients were invited to participate, of whom 54 accepted; 34 patients were selected, as they had reached their final height. Anthropometric, blood glucose, insulin, and lipid and hormonal profile (LH, FSH, estradiol, 17α-OH-progesterone, androstenedione, dehydroepiandrosterone sulfate, testosterone) data were obtained, the HOMA-IR index was calculated, and pelvic ultrasonography was performed. To characterize polycystic ovary syndrome and metabolic syndrome, the Rotterdam and International Diabetes Federation criteria, respectively, were used. Data were analyzed according to measures of dispersion, frequency and correlations of interest.

**RESULTS:**

The age of the participants ranged from 15.2 to 28.2 years/months; 23.5% of the patients were overweight, 11.8% were obese, 29.4% had a large waist circumference, and 8.8% were hypertensive. None of the patients had altered glucose levels, and insulin levels and HOMA-IR were elevated in 29.4% and 38.2% of the participants, respectively; 14.7% of the patients exhibited *acanthosis nigricans*. The lipid profiles of the participants were variable, and one patient (2.9%) had metabolic syndrome. Polycystic ovary syndrome was found in 41.2% of patients.

**CONCLUSION:**

The percentage of patients with polycystic ovary syndrome who also had overweight, obesity and insulin resistance corroborates the literature data about the need for follow-up aiming at interventions, especially for conditions associated with cardiometabolic risk.

## INTRODUCTION

Some children develop isolated and idiopathic premature adrenarche (PA), which is defined as the onset of androgenic signs before 8 years of age in girls and 9 years of age in boys, in the absence of true puberty, steroidogenic enzymatic defects and virilizing tumors. PA corresponds to the presence of androgen action signs, including adult body odor, oily skin and pubic hair growth. Precocious pubarche (PP) more specifically refers to the early appearance of pubic and/or axillary hair in these age groups 1,2.

The incidence of PA varies among different ethnic populations, with reports of incidence rates varying from 5% to 30% and a higher incidence in non-Hispanic black and white children; PA has been reported to be up to 10 times more common in girls than in boys 3,4.

The etiology of PA is not well understood. It is postulated that its onset arises as a result of the early activation of the maturation of the adrenal reticular zone, which is associated with increased production of adrenal androgens, and as a result of increased peripheral conversion of adrenal androgens to testosterone and dihydrotestosterone 1,2.

The diagnosis of PA requires the exclusion of other causes of excess androgens, such as congenital adrenal hyperplasia, androgen-producing tumors, precocious puberty or exogenous sources of androgens. In general, obtaining a thorough history and performing a physical examination and baseline measurement of adrenal androgens and eventually gonadotropins is sufficient to make a differential diagnosis between idiopathic PA and these conditions 1,2,5.

Until the late 1990s, PA was considered a benign variant of pubertal development, with no need for special monitoring. Subsequently, several studies have correlated PA with metabolic syndrome (MS) components and with several other disorders, including functional adrenal hyperandrogenism 5,6.

Ibáñez et al., in follow-up studies of 35 teenage girls with PA, reported a spectrum of endocrine-metabolic abnormalities in 16 girls (45%); these abnormalities included central adiposity, even in the absence of obesity; hirsutism; menstrual disorders; and polycystic ovary syndrome (PCOS). Furthermore, hyperinsulinemia, insulin resistance, increased levels of androgens associated with patterns of ovarian and adrenal hyperresponsiveness, and adrenocorticotropic hormone (ACTH) and gonadotropin-releasing hormone (GnRH) agonist stimulation were found. In addition, a shift in the activity of adrenal enzymes was observed, with the accretion of 17,20-lyase and 17-hydroxylase activities, resulting in elevated levels of DHEA and DHEA sulfate (DHEA-S) for chronological age 5,7.

Miller 9 suggested a “unified theory of adrenarche, PCOS and hyperinsulinemia”, and Ibáñez et al. 4 suggested that the underlying mechanism may be abnormal regulation or increased expression of the CYP17A1 androgen synthesis enzyme in the adrenals and ovaries 5. The researchers suggested precocious treatment with metformin, an insulin-sensitizing drug, and lifestyle changes aimed at reducing hyperinsulinemia, as some authors have suggested as a treatment for PCOS 10-13.

In view of the literature reports, and considering that PA predisposes patients to hormonal abnormalities, an increased probability of developing PCOS, and conditions related to cardiometabolic risk, we intend to assess if such abnormalities are present in our environment and in patients who were previously treated and followed up at the Endocrinology Outpatient clinic for PA.

## METHODS

Data collection occurred between March and December 2016, after the project was approved by the Human Research Ethics Committee of Universidade Federal do Triângulo Mineiro. Data were collected while patients were being treated at the Gonads and Adrenal Outpatient Clinic of the Discipline of Endocrinology and Metabology, after signing the free and informed consent form.

A total of 130 medical records were obtained from the files of patients with a diagnosis of PA. After analysis, eight patients were excluded because they had central precocious puberty. One hundred and twenty-two patients were invited by letter for re-evaluation, and only 54 accepted the invitation. After the initial consultation, 20 patients who had not yet reached menarche or had not reached their final height were excluded; thus, a total of 34 patients were selected for study inclusion.

Anamnesis and a physical examination were performed as well as an anthropometric evaluation that included weight, height, body mass index (BMI), abdominal circumference (AC), blood pressure (BP), pubertal staging, the presence of acne and *acanthosis nigricans*, and body hair distribution to identify hirsutism according to the Ferriman-Gallwey score 14.

Weight was measured using a digital scale (Filizola^®^, São Paulo, Brazil) with a capacity of up to 300 kg. Height was measured using a wall stadiometer (Toneli^®^, Criciúma, Brazil, model E 150 A) while the participant was in the standing position, barefoot, with feet placed together. BMI was calculated by dividing the weight by the height squared. The BMI Z-score (Z-BMI) was used to classify the nutritional status of those younger than 19 years, and the Z-BMI was standardized according to the World Health Organization (WHO) criteria 15 and was calculated with WHO-Anthro Plus 2007 (Geneva, Switzerland). The WHO classification was also used for patients older than 19 years. The AC was measured using an inextensible measuring tape graduated in millimeters at the midpoint between the lower border of the last rib and the upper border of the iliac crest with the patient in the standing position. For girls up to 17 years old, the AC was considered to be high when its value was ≥ the 90^th^ percentile 16. For women older than 17 years, the AC was considered to be high when its value was ≥80 cm, according to the International Diabetes Federation (IDF) consensus 17.

The homeostatic model assessment for insulin resistance (HOMA-IR) index was calculated according to the formula: Fasting glucose (mmol/L) x Fasting insulin (µU/mL)/22.5 18. The reference for the HOMA-IR index for the Brazilian population, according to the BRAMS study for individuals ≥18 years is as follows: normal <2.7 and elevated ≥2.7 19. According to the Brazilian Diabetes Society in its 2015-2016 guidelines and based on a study by Stern et al. 20, the presence of insulin resistance should be evaluated based on three parameters: 1) BMI >28.9 kg/m^2^, 2) HOMA-IR >4.6 and 3) BMI >27.5 kg/m^2^ associated with HOMA-IR >3.6, and for those younger than 18 years of age, the HOMA-IR cut-off value of 3.43 is considered for Tanner 4 and 5 adolescents 21.

The presence of *acanthosis nigricans* was verified through visual assessment of the skin in regions such as the neck, armpits, and inguinal region; the presence of acne was also verified through visual assessment of the skin. Hirsutism was assessed using the Ferriman-Gallwey score 14. BP was measured using a stethoscope and sphygmomanometer on the right arm, with the patient in the sitting position. BP assessment was carried out according to the 7^th^ Brazilian Guideline of Systemic Arterial Hypertension 22. Pubertal staging was evaluated visually and classified according to the Marshal and Tanner criteria 23.

The following laboratory tests were performed after a 10-12 hour fasting period: fasting glucose (FG), basal insulin (BI), glycated hemoglobin (HbA1c), total cholesterol (TC), triglycerides (TGs), HDL-cholesterol (HDL-c), LDL-cholesterol (HDL-c), non-HDL-cholesterol (NHDL-c), VLDL-cholesterol (VLDL-c), luteinizing hormone (LH), follicle-stimulating hormone (FSH), estradiol, thyroid-stimulating hormone (TSH), free thyroxine (free T4), total testosterone, 17α-OH-progesterone (17-OH-P), androstenedione (Δ4-A), and dehydroepiandrosterone sulfate (DHEAS). The fasting glucose/insulin (FG/I) ratio, the HOMA-IR index, and the triglyceride/HDL ratio (TG/HDL) were also calculated.

FG and HbA1c were measured using the automated colorimetric method, whereas TC, its fractions and TGs were measured using the esterase cholesterol colorimetric enzymatic method. The lipid profile evaluation was performed according to the V Brazilian Guideline on Dyslipidemias and Atherosclerosis Prevention 24. The FG/I ratio was considered altered when it was >0.30 25. The TG/HDL ratio was considered altered when it was >3.5 26. HbA1c values between 5.7 and 6.4% indicated individuals at high risk for the development of diabetes, and levels ≥6.5 indicated the presence of diabetes 27.

To characterize MS in patients in the age range of 10-17 years, the IDF criteria with adapted blood pressure levels were used, considering an AC ≥90^th^ percentile associated with two other criteria: FG ≥100 mg/dL or DM, TGs ≥150 mg/dL, HDL-c <40 mg/dL, or systolic or diastolic BP ≥95^th^ percentile according to gender, age and height 17.

To characterize MS in adult women, the IDF criteria were used, with AC ≥80 cm associated with two variables: TGs ≥150 mg/dL or triglyceride-lowering treatment, HDL-c <50 mg/dL, systolic BP ≥130 mmHg or diastolic BP ≥85 mmHg or hypertension treatment, or FG ≥100 mg/dL or DM2 17.

LH, FSH, DHEAS, testosterone, insulin, TSH, and free T4 levels were measured using Elecsys Assay commercial kits (Roche Diagnostics GmbH, Mannheim, Germany) according to the electrochemiluminescence method (ECLIA) and were analyzed in a Cobas 6000 and 601 automated system (Roche Diagnostics). The minimum detection value was 0.1 mUI/mL for both LH and FSH. The inter- and intra-assay coefficients of variation (CVs) of LH are both approximately 2%. FSH has inter- and intra-assay CVs of up to 4.5% and 2.8%, respectively. The minimum detection values for DHEAS, testosterone, insulin, TSH, and free T4 were 0.1 µg/dL (interassay CV: 3.6% and intra-assay: 2.8%), 2.5 ng/mL (interassay CV: 8.4% and intra-assay: 4.7%), 0.2 µU/mL (interassay CV: 2.6% and intra-assay: 1.9%), 0.000014 mUI/L (interassay CV: 7.2% and intra-assay: 3.0%) and 0.04 ng/dL (interassay CV: 6.3% and intra-assay: 5.0%), respectively. Estradiol was also measured using the Elecsys Estradiol III Assay (Roche Diagnostics GmbH, Mannheim, Germany) according to the ECLIA and was analyzed by the same automated system as was used for the abovementioned hormones. The method's lower limit value is 5 pg/mL, and the inter and intra-assay CVs were up to 10.6% and 6.7%, respectively.

The measurement of 17-OH-P was performed by radioimmunoassay using NEXGEN® equipment (Seoul, South Korea), and the minimum detection value of the method was 0.052 ng/L, with inter- and intra-assay CVs up to 23.0% and 11.8%, respectively. Androstenedione was measured by chemiluminescence with SIEMENS IMMULITE 2000® equipment (Germany); the minimum detection value of the method is 0.3 ng/mL, with inter- and intra-assay CVs up to 13.5% and 11.6%, respectively.

For the diagnosis of PCOS in adolescents, the presence of three criteria was considered: clinical and biochemical hyperandrogenism, oligoanovulation and enlarged or micropolycystic ovaries identified by an ultrasound assessment; and irregular menstrual cycles that persisted for at least 2 years after menarche 28. In adult women, two diagnostic consensuses were used, including the Rotterdam consensus 29, which diagnoses PCOS when the patient has two of the following three criteria: clinical and biochemical hyperandrogenism, oligoanovulation/anovulation and polycystic ovaries identified by an ultrasound assessment. Polycystic ovaries are considered based on the presence of at least 12 follicles measuring 2 to 9 mm and ovarian volume >10 mL. the other consensus that was used was the Androgen Excess Society (AES) Consensus 30, which requires clinical and biochemical hyperandrogenism and ovarian dysfunction and/or polycystic ovaries identified by an ultrasound assessment. The latter includes a new technology that increased the threshold of the ovarian follicle count to 25 from 2 to 9 mm or ovarian volume >10 mL.

This is a descriptive study, and the variables of interest were expressed through central and dispersion measures. The Shapiro-Wilks test was used to verify the normal distribution of the variables. Correlations between numerical variables were analyzed by Pearson's or Spearman's tests, depending on parametric or nonparametric distribution, respectively; a *p* value ≤0.05 was considered significant. The calculations were performed using the IBM software SPSS, STATISTIC version 20 (New York, USA).

## RESULTS

### Initial data of patients with premature adrenarche

The retrospective analysis of the selected patients' files showed that at the first consultation, the patients ranged from 10 months to 10 years and seven months of age (median 7; 1). The mean weight Z-score was 0.3±1.7, and the mean height Z-score was 0.3±1.3. The mean (Z-BMI) was 0.5±1.4. Twenty-three patients had normal weight, five were overweight and six were obese.

The pubertal stage was B1P2 in 31 girls and B1P3 in three girls. None of the girls had arterial hypertension. The FG level was 85.4±12.3 mg/dL, and only three participants had alterations in FG, but none of the participants had diabetes. Regarding the lipid profile, the mean TC levels ranged from 111.5 to 254.2 (median: 142.0) and were elevated in six patients. The mean TG levels were 100.4±44.8 mg/dL; the TG levels were borderline in six patients and elevated in three patients. The mean serum levels of HDL-c were 52.4±16.0 mg/dL and were low in four participants. The mean serum LDL-c levels were 86.5±26.2 mg/dL and were borderline in two patients and elevated in one patient.

Previous hormonal data showed TSH concentrations of 2.2±1.2 mIU/L. Only one patient showed subclinical hypothyroidism. The mean estradiol level was 24.0±10.7 pg/mL. The median LH level was 0.6 mIU/mL (minimum: 0.1, maximum: 3.0), and the mean FSH concentration was 1.2±0.9 mIU/mL. The median testosterone level was 20.0 ng/dL (minimum: 1.0, maximum: 67.3), and three patients had elevated testosterone levels. The median concentration of DHEAS was 30.0 μg/dL (minimum: 2.0, maximum: 135.0), and one patient had elevated DHEAS. The mean serum level of 17-OH-P was 1.1±0.6 ng/mL; two patients had levels >2.0 ng/dL and were submitted to the ACTH analog test (Cortrosyn, 250 μg iv), showing normal values. The average birth weight was 2940.3±574.2 grams; the birth weight was less than 2400 grams in six patients, of which three were considered small for gestational age (SGA).

### Current data

In the 34 patients selected, the median age was 20 years and 3 months (minimum: 15.2; maximum: 28.2). The mean weight was 63.8±14.5 kg, and the mean height was 162.8±7.3 cm. The mean BMI was 24.0±4.9 kg/m^2^. One patient (2.9%) had low weight, 21 (61.8%) had normal weight, eight (23.5%) were overweight and four (11.8%) were obese.

The mean AC was 76.0±10.6 cm, and ten patients (29.4%) had a large AC. The mean systolic BP (SBP) was 112.5±12.3 mmHg, and the mean diastolic BP (DBP) was 73.2±9.0 mmHg; three patients (8.8%) were categorized as hypertensive. The Ferriman-Gallwey score was assessed in 20 patients, and 11 (55%) showed hirsutism (score ≥8). Acne occurred in 13 patients (38.2%), and *acanthosis nigricans* occurred in five (14.7%). Patients reported menarche at an average age of 11 years and 10 months (minimum: 10.0, maximum: 14.0). The clinical and anthropometric data are shown in Table 1.

Metabolic data are shown in Table 2. The mean FG was 79.7±9.4 mg/dL, and none of the patients had alterations. The median HbA1c was 6.0% (minimum: 4.3%, maximum: 6.1%), and two patients (5.9%) had HbA1c values indicative of glucose intolerance. The mean BI levels were 12.6±5.6 mIU/mL. Hyperinsulinemia was observed in ten patients (29.4%). The mean insulin/FG (I/FG) ratio was 0.2±0.1, and it was considered altered in two patients (5.9%). The mean HOMA-IR index was 2.4±1.2. Of the patients older than 18 years, eight (39.0%) had insulin resistance according to Geloneze et al. 19, while six (28.6%) met the criteria of Stern et al. 20. For those younger than eighteen years, using the criteria of Gárcia-Cuartero et al. 21, five patients (38.5%) had an increased HOMA-IR index.

The mean TC level was 168.9±24.8 mg/dL, and TC was considered elevated in five patients (14.7%). The mean TG concentration was 85.6±28.7 mg/dL and was elevated in two patients (5.9%). The mean HDL-c concentration was 58.3±14.9 mg/dL, and five patients had low HDL-c levels. The mean LDL-c level was 93.1±24.3 mg/dL, and the LDL-c level was normal in all patients. The mean level of NHDL-c was 110.6±26.6 mg/dL and was considered high in only one patient (2.9%). The mean TG/HDL ratio was 1.6±1.0, and three patients (8.8%) had an increased ratio 26. Only one patient (2.9%), aged 18 years and 3 months, met the criteria for MS.

The hormonal data are shown in Table 3. The mean TSH levels were 2.6±1.5 mIU/L. The TSH level was low in two patients (5.9%), normal in 29 (85.3%), and elevated in 3 (8.8%). The mean level of free T4 was 1.2±0.2 ng/dL. Four patients (11.8%) had low free T4 levels, and one (8.8%) had a discreetly increased free T4 level.

It was proposed that blood samples for hormonal assessment should be collected in the early follicular phase; however, three patients (8.8%) did not meet our request. The mean LH level was 6.0±4.2 mIU/mL, and the mean FSH level was 4.4±1.9 mIU/mL. The mean LH/FSH ratio was 1.3±0.8, and it was ≥2 in nine patients (26.5%).

The mean testosterone levels were 29.6±14.0 ng/dL and were increased in four patients (11.8%) and normal in 88.2%. The mean DHEAS level was 193.3±99.9 µg/dL and was analyzed according to age: in the patients younger than 19 years, the DHEAS levels were normal in 14 (87.5%) and elevated in two (12.5%); among those aged 20 to 24 years, the levels were normal in all cases, and in those aged 25 to 34 years, the levels were normal in six patients (85.7%) and elevated in one patient (14.3%).

The mean level of 17-OH-P was 1.6±2.0 ng/mL. Eight patients (23.5%) had elevated levels. These patients were submitted to the ACTH analog test (Cortrosyn, 250 µg iv), and the resulting values ruled out adrenal hyperplasia 1. The median Δ4-A levels were 2.1 ng/mL (minimum: 0.3 ng/mL, maximum: 4.2 ng/mL), and two patients (5.9%) had high levels.

The median volume of the right ovary was 7.2 mL (minimum: 1.2 mL, maximum: 150.0 mL), and 12 patients (35.3%) showed an increased volume. The median left ovary volume was 7.5 mL (minimum: 1.5 mL, maximum: 96.0 mL), and eight (23.5%) showed an increased volume. Eight patients (23.5%) had ovaries with a micropolycystic aspects.

Among patients younger than 19 years, three (18.7%) met the criteria for PCOS. Among patients older than 19 years, eleven patients (62.0%) had PCOS according to the Rotterdam consensus criteria. Using the AES Consensus criteria, the same eleven patients (62.0%) had PCOS, showing there was a concordance between the two consensuses used. Overall, 14 patients (41.2%) met the criteria for PCOS.

Correlations between clinical and metabolic data were analyzed: BMI showed a positive and weak correlation with the insulin/glucose ratio (r=0.362, *p*<0.05) and with the HOMA-IR index (r=0.358, *p*<0.05). The AC showed a positive and moderate correlation with BI (r=0.526, *p*<0.01) and a weak correlation with HbA1c (r=0.352, *p*<0.05), triglycerides (r=0.544, *p*<0.01), the insulin / glucose ratio (r=0.471, *p*<0.01), and HOMA-IR (r=0.613, *p*<0.01). SBP presented a moderate positive correlation with HbA1c (r=0.457, *p*<0.01) and a weak positive correlation with the HOMA-IR index (r=0.338, *p*<0.05). No significant correlations were observed between the reported clinical and hormonal data.

There was a positive and weak correlation between birth weight and the current metabolic data with the HOMA-IR index (r=0.395, *p*<0.05). Among patients for whom it was possible to calculate target height (n=18), only four did not reach it. The correlation between target height and final height was positive and moderate (r=0.588; *p*=0.001). The current BMI showed a correlation with the initial BMI (r=0.713, *p*=0.001) and with the Z-score of the initial BMI (r=0.550, *p*<0.001). Correlations were observed between current and initial 17-OH-P levels (r=0.496, *p*<0.05).

## DISCUSSION

The present study suggests, as previously reported in the literature 5,6, that PA is not a “variant of normal” condition, as it has been classically considered, and careful attention should be directed to some aspects during the follow-up of these children.

Despite the small percentage of MS in the patients (2.9%), abnormalities related to cardiometabolic risk were present as overweight (23.5%) and obesity (11.8%), and a significant correlation was found between the current BMI and the initial BMI (r=0.713, *p*<0.01) and the initial (Z-BMI) (r=0.550, *p*<0.01). These data indicate the need for preventive measures against obesity in children with PA. According to the Brazilian Guidelines for Obesity 30, an obese child will remain obese in adulthood, and the likelihood ranges from 20-50% before puberty to 50-70% after puberty.

Systemic Arterial Hypertension (SAH) was observed in 8.8% of the patients, whereas the prevalence of SAH in Brazil in individuals aged between 18 and 29 years is 2.8% and ranges from 3 to 5% in the pediatric age range 31. BP shows normal circadian fluctuations; adolescents with PCOS and abnormal glucose tolerance have an interruption in these fluctuations, and this may be a precursor for the development of arterial hypertension in this population 32.

When analyzing the glucose metabolism data, only 5.88% of the patients showed HbA1c levels indicative of glucose intolerance, and FG was normal in all of the patients; however, as reported in the literature, several factors demonstrated insulin resistance, such as *acanthosis nigricans*, which occurred in 14.7% of the patients; fasting hyperinsulinemia, which occurred in 29.4% of the patients; altered insulin/glucose ratio, which occurred in 5.9% of the patients; increased HOMA-IR index, which occurred in 38.2% of the patients; and an altered TG/HDL ratio, which occurred in 8.8% of the patients, corroborating the findings of other authors who associated PA with these conditions 1,2,4-6.

The lipid profile was adequate in most patients, but 14.7% of the patients had high TC levels, and 5.88% had high triglycerides, whereas 14.7% had low HDL-c levels. These are young patients who already exhibit a lipid alteration pattern associated with MS characterized by decreased HDL-c and elevated triglyceride levels 33, suggesting the need for patient guidance in relation to these aspects.

Positive and significant correlations between adiposity indexes and insulin resistance markers are expected since overweight/obese patients have insulin resistance; therefore, a causal association between these variables is expected. The same concept applies to the BP analysis, which showed a moderate positive correlation with HbA1c (r=0.457, *p*<0.01) and with the HOMA-IR index (r=0.338, *p*<0.05), suggesting that HOMA-IR and HbA1c could be markers of cardiometabolic risk.

According to Ibáñez et al. 4,5, children with intrauterine growth retardation, or those who are SGA, are more likely to show an increase in growth factors, such as IGF-1 and hyperinsulinemia, as compensatory changes to catch up in terms of weight and height, but as a consequence, they can develop PA, insulin resistance, and MS during their life span. The present study showed only three patients (8.8%) with such conditions, and a significant positive correlation was found between birth weight and the HOMA-IR index, suggesting that being overweight at birth may also be associated with the risk of obesity and insulin resistance in adults.

Another group of abnormalities that was found was related to androgenic action and gonadal function. Hirsutism assessment using the Ferriman-Gallwey score showed limitations because many patients had shaved before the consultation, allowing the analysis in only 20 patients, and the hirsutism status was altered in 55% of them. Hirsutism affects 5 to 10% of women, and therefore, the percentages found in the present study were higher than those in the overall population 34. However, no statistically significant correlation was found between the Ferriman-Gallwey score and circulating androgen levels, corroborating the literature data showing that hirsutism is associated with a localized phenomenon of increased sensitivity of hair follicles to circulating androgen levels, which are more intensely metabolized at the receptor sites, which is one of the explanations of PA pathophysiology and hirsutism 34.

When analyzing the hormonal data, an LH/FSH ratio suggestive of PCOS (>2) was found in 26.5% of the patients, and androgen concentrations were in the normal range in the majority of the patients (11.8% had high testosterone levels, 8.8% had elevated DHEAS levels). The most evident alteration was observed in the 17-OH-P level, which was elevated in 23.5% of the patients. Although congenital adrenal hyperplasia was ruled out, Ibanez et al. reported the presence of functional ovarian and adrenal disorder in patients with PA 4,7,8. Correlating the hormonal data from the start of the follow-up with the current data, a positive and significant correlation can be observed between the initial levels of 17-OH-P (*p*<0.05) and the current levels, showing that this alteration was maintained during the assessment period.

The presence of PCOS was found in 41.2% of the patients in the sample studied, which is higher than the figure reported by March et al. 35, who found a prevalence of 6-10% in a general population, and in the context of the abnormalities observed, PCOS was described as being associated with overweight/obesity, insulin resistance and MS. As suggested, the pathophysiology of PCOS is complex, being the common final pathway of a variety of metabolic and hormonal expressions, which occur in addition to the genetic and epigenetic factors involved 29.

The present study has some limitations: a small number of patients were analyzed; the 122 patients who were attended at the Pediatric Endocrinology outpatient clinic should have been followed up at the Gonads and Adrenal outpatient clinic; however, surprisingly, they had to be called, and only 44.3% of the patients agreed to participate in the study. This loss of follow-up limited the study to two cross-sections at the beginning of the study period and at the present time; the clinical hirsutism defined by the Ferriman-Gallwey score could not be determined in all patients because they shaved prior to the consultation. The strength of the study is the demonstration that patients with PA should undergo long-term follow-up aiming for the early intervention of cardiometabolic risk factors and SOP.

## CONCLUSION

The percentage of individuals with PCOS in the patients with PA was greater than that in the overall population, which demonstrates that patients with PA share some pathophysiological elements with PCOS, such as hyperinsulinemia, insulin resistance, functional adrenal alterations, and other elements that are still poorly defined within the complex pathophysiology of both conditions. PA should not be considered a “variant of normal” child development but rather a clinical event that deserves attention and early intervention in terms of metabolic risk factors, PCOS and MS during a long-term and careful follow-up.

## AUTHOR CONTRIBUTIONS

Ribeiro FA and Borges MF developed the project, coordinated the work and wrote the manuscript. Resende EA and Palhares HM guided the patients according to the methods reported in this work and guided the careful treatment and follow-up of the patients. Tomé JM and Silva AP collected the patient records and plotted the data in the Excel tables, they also performed the statistical analysis and configured the tables.

## Figures and Tables

**Table 1:** Current clinical and anthropometric data of the 34 patients assessed.

Assessed Parameters	x̄*±SD* or Median^#^	Minimum	Maximum
Age (years; months)	20.3	15.2	28.2
Weight (kg)	63.8±14.5	42.5	105.9
Height (cm)	162.8±7.3	151.0	179.0
BMI (kg/m^2^)	24.0±4.9	16.5	36.6
AC (cm)	76.0±10.6	60.0	106.5
SBP (mmHg)	112.5±12.3	90.0	150.0
DBP (mmHg)	73.2±9.0	60.0	90.0
Ferriman-Gallwey score (n=20)	9.0±5.7	1	20
Menarche (years; months)	11.1	10.0	14.0

Source: The author, 2017.

# according to normal or normal distribution

BMI (body mass index): >19 years: low weight <18; normal weight: 18-24.9; overweight: 25-29.9; obesity >30. BMI (kg/m^2^) <19 years; normal weight -2≤ z-BMI <+1; overweight +1 ≤ z-BMI <+2; obesity z-BMI ≥+2

AC (abdominal circumference) >17 years: normal <80; altered >80; AC <17 years: high ≥90^th^ percentile

SBP (systolic blood pressure) and DBP (diastolic blood pressure) ≤17 years of age: hypertensive ≥95^th^ percentile

SBP (systolic blood pressure) >17 years: Normal ≤120; Prehypertension: 121-139; Hypertension ≥140. DBP (diastolic blood pressure) >17 years: normal ≤80; prehypertension: 81-89; hypertension ≥90

Ferriman-Gallwey score: <8 normal; >8 hirsutism

**Table 2:** Current metabolic data of the 34 patients assessed.

Assessed Parameters	x̄*±SD* or Median^#^	Minimum	Maximum
FG (mg/dL)	79.7±9.4	57.9	93.3
HbA1c (%)	6.0	4.3	6.1
BI (µUI/mL)	12.6±5.6	2.0	25.8
I/FG ratio	0.2±0.1	0.03	0.4
HOMA-IR	2.4±1.2	0.02	4.7
TC (mg/dL)	168.9±24.8	121.6	226.4
TGs (mg/dL)	85.6±28.7	33.0	143.0
HDL-c (mg/dL)	58.3±14.9	30.0	92.0
LDL-c (mg/dL)	93.1±24.3	39.2	144.2
Non-HDL-c (mg/dL)	110.6±26.6	60.6	164.4
VLDL-c (mg/dL)	18.0±5.7	6.6	28.6
TG/HDL ratio	1.6±1.0	0.4	4.8

Source: the author, 2017.

# according to normal or nonnormal distribution

FG (Fasting glucose): normal <100; altered ≥100, diabetes ≥126 mg / dL

BI (basal insulin): normal <15; altered ≥15

FG/I: fasting glucose/insulin ratio

HbA1c (glycated hemoglobin): normal <5.7; 5.7-6.4 = increased risk for diabetes, ≥6.5, diabetes

HOMA - IR: adults >18 years = normal until 2.7; adolescents Tanner IV and V = up to 3.43

TC (total cholesterol) >20 years: desirable <200; borderline: 200-239; high >240

TC (total cholesterol) 2-19 years: desirable <150; borderline: 150-169; high ≥170

TGs (triglycerides) >20 years: desirable <150; borderline: 150-200; high: 200-499; very high ≥500

TGs (triglycerides) 2-19 years: desirable <100; borderline: 100-129; high ≥130

HDL-c (HDL-cholesterol) >20 years: desirable >60; low <40

HDL-c (HDL-cholesterol) 2-19 years: ≥45

LDL-c (LDL-cholesterol) >20 years: optimal <100; desirable: 100-129; borderline: 130-159; high: 160-189; very high ≥190

LDL-c (LDL-cholesterol) 2-19 years: desirable <100; borderline: 100-129; high ≥130

Non-HDL-c (non-HDL-cholesterol) >20 years: optimal <130; desirable: 130-159; high: 160-189; very High ≥190

VLDL-c (VLDL-cholesterol) >20 years: desirable <30; High >30

TG/HDL (triglyceride/HDL-cholesterol ratio): altered >3.5

FG/I (fasting glucose/insulin ratio) >0.30 altered

**Table 3:** Current hormonal data of the 34 patients assessed.

Assessed Parameters	x̄*±SD* or Median^#^	Minimum	Maximum
TSH (mUI/L)	2.6±1.5	0.01	6.8
Free T4 (ng/dL)	1.2±0.2	0.7	1.7
LH (mUI/mL)	6.0±4.2	0.1	16.7
FSH (mUI/mL)	4.4±1.9	0.3	7.8
LH/FSH	1.3±0.8	0.3	3.0
E2 (pg/mL)	51.5	5.0	244.3
TESTOSTERONE (ng/dL)	29.6±14.0	9.0	68.7
DHEAS (µg/dL)	193.3±99.9	45.4	429.9
17α-OH-Progesterone (ng/mL)	1.6±2.0	0.2	5.7
Δ4-A (ng/mL)	2.1	0.3	4.2

Source: the author, 2017.

# according to normal or nonnormal distribution

TSH (thyroid-stimulating hormone) normal: 0.27-4.20

Free T4 (free thyroxin) normal: 0.93-1.70

E2 (estradiol): follicular phase: 12.5-166; ovulatory phase: 85.8-498; luteal phase: 43.8-211

Testosterone, women: 20-49 years: 8.40-48.1

DHEAS (dehydroepiandrosterone sulfate): women 15-19 years: 65.1-368; 20-24 years: 148-407; 25-34 years: 98.8-340

17α-OH-progesterone (17 α hydroxyprogesterone) women: follicular phase: 0.11-1.08; luteal phase: 0.95-5.0

Δ4-A (androstenedione): women: 0.3-3.7

LH (luteinizing hormone): women: follicular phase: 2.4-12.6; ovulatory phase: 14.0-95.6; luteal phase: 1.0-11.4

FSH (follicle-stimulating hormone): women: follicular phase: 3.5-12.5; ovulatory phase: 4.7-21.5; luteal phase: 1.7-7.7

LH/FSH (luteinizing hormone/follicle-stimulating hormone ratio) <2.0 normal; >2.0 suggestive of PCOS
